# Stabilization of Transport Properties in Thin Nonstoichiometric La_1−x_Sr_x_Mn_y_O_3_ Films via Accelerated Aging for Magnetic Field Sensors

**DOI:** 10.3390/s26092711

**Published:** 2026-04-28

**Authors:** Vakaris Rudokas, Mykola Koliada, Voitech Stankevic, Skirmantas Kersulis, Vilius Vertelis, Sonata Tolvaišienė, Martynas Skapas, Milita Vagner, Valentina Plausinaitiene, Nerija Zurauskiene

**Affiliations:** 1 Department of Functional Materials and Electronics, Center for Physical Sciences and Technology, Sauletekio Ave. 3, 10257 Vilnius, Lithuania; vakaris.rudokas@ftmc.lt (V.R.); mykola.koliada@ftmc.lt (M.K.); voitech.stankevic@ftmc.lt (V.S.); skirmantas.kersulis@ftmc.lt (S.K.); vilius.vertelis@ftmc.lt (V.V.); martynas.skapas@ftmc.lt (M.S.); milita.vagner@ftmc.lt (M.V.); valentina.plausinaitiene@ftmc.lt (V.P.); 2 Faculty of Electronics, Vilnius Gediminas Technical University, 10223 Vilnius, Lithuania; sonata.tolvaisiene@vilniustech.lt; 3 Faculty of Chemistry and Geosciences, Vilnius University, 03225 Vilnius, Lithuania

**Keywords:** polycrystalline manganite films, magnetic field sensors, colossal magnetoresistance, aging effects

## Abstract

Magnetic sensors based on the colossal magnetoresistance (CMR) effect in manganite thin films are promising for high-field measurements due to their wide operating range, low magnetoresistance anisotropy, and ability to function without full saturation at extremely high magnetic fields. However, the long-term stability of their transport properties remains a key challenge for practical sensor applications. In this work, accelerated aging of nanostructured La_1−x_Sr_x_Mn_y_O_3_ thin films was investigated for two manganese compositions: nominally stoichiometric (*y* = 1.05) and Mn-excess (*y* = 1.15). The electrical resistivity and magnetoresistive properties strongly depended on the manganese content and substrate type. Accelerated aging was induced by annealing at 100 °C in an argon atmosphere, and the evolution of the transport properties was analyzed using a stretched-exponential relaxation model. The analysis of the extracted parameters indicated defect-related mechanisms governing transport stability. It was found that despite the increase in resistivity during thermal treatment, the magnetoresistance changes were insignificant. The results provide insights into the aging behavior of nonstoichiometric manganite films and offer guidance for optimizing stabilization procedures in CMR-based magnetic field sensors.

## 1. Introduction

Polycrystalline thin films of lanthanum strontium manganite, La_1−x_Sr_x_MnO_3_ (LSMO), are of significant technological interest due to their colossal magnetoresistance (CMR) across wide temperature and magnetic field ranges [1,2,3,4]. These properties make them promising for magnetic field sensing in extreme environments, including pulsed-field magnets, electromagnetic railguns, flux compression generators, plasma diagnostics, and beam-guiding systems [5,6,7]. Based on their characteristics, a family of CMR-B-scalar sensors using nanostructured LSMO films grown on polycrystalline substrates has been developed [8]. Compared with conventional Hall, inductive *B*-dot, or Faraday-effect optical sensors, these devices can measure magnetic fields up to megagauss amplitudes, are insensitive to field direction, and operate over extremely short time scales and wide temperature ranges [5,9].

The CMR sensing principle relies on the magnetic field dependence of film resistivity. However, the ambient temperature strongly affects both the initial resistance and sensitivity to magnetic fields, complicating accurate field measurements [10,11]. Although temperature compensation methods have been proposed [8], the practical use of these sensors is limited by the long-term stability of their functional properties. Similar reliability challenges have been reported across a range of functional oxide-based materials, where aging effects are governed by defect migration and grain-boundary (GB) processes. For instance, studies on nanostructured spinel-oxide thick films demonstrated that thermally induced aging results in diffusion-driven redistribution of material along grain boundaries and contact regions, producing resistance drift that follows non-exponential relaxation kinetics [12]. Investigations on epitaxial thin films further showed that temperature-driven defect engineering, particularly involving oxygen vacancies and interfacial diffusion processes, has a decisive influence on both the magnetic and transport properties [13]. These findings highlight the critical role of defect dynamics in manganite systems and underscore the importance of controlled thermal treatment for stabilizing their functional characteristics. Comparable conclusions were also reached in comprehensive reliability studies on MgO-based tunneling magnetoresistance sensors, where accelerated electrical, thermal, and environmental stresses revealed defect-controlled aging mechanisms as the dominant factor limiting long-term sensor stability [14].

Aging in nanostructured manganite films is governed by structural relaxation processes involving oxygen vacancy migration, defect redistribution, and changes in magnetic ordering [15]. These films can be considered assemblies of randomly oriented nanocrystallites separated by structurally and magnetically disordered grain boundaries, which act as pathways for defect diffusion and therefore strongly influence resistance stability [16]. The kinetics of such monotonically rising and decaying relaxation processes in disordered systems generally require three fitting parameters. However, such processes are commonly described using the two-parameter stretched-exponential function exp[−(*t*/*τ*)*^k^*], where *τ* is the characteristic relaxation time and *k* is the stretching exponent [16,17]. This approach has previously been successfully applied to describe accelerated aging induced by thermal annealing of LSMO manganite films [18].

Despite the progress in stabilizing the transport properties of stoichiometric films, particular attention has recently been directed toward manganite thin films with intrinsic compositional nonstoichiometry [19], especially those with manganese excess. Such films are of significant interest because increasing the manganese content (*y* = Mn/(La + Sr) > 1) in La_1−x_Sr_x_Mn_y_O_3_ leads to a systematic shift in the metal–insulator transition temperature (*T*_m_) toward higher values, enabling operation at elevated temperatures. This property is crucial for high-temperature sensor applications, where conventional stoichiometric films are limited by their lower *T*_m_. In addition, Mn-excess compositions often exhibit reduced resistivity in the vicinity of *T*_m_, which is beneficial for minimizing noise and improving impedance matching in pulsed-field sensing systems [9,20]. It was shown by Marozau et al. [21] that deviations from the ideal stoichiometry—specifically lanthanum deficiency (i.e., Mn excess)—strongly modifies transport and magnetic transitions via the Mn^3+^/Mn^4+^ ratio of LaMnO_3_. However, despite these advantages, the influence of accelerated aging—known to stabilize resistance in stoichiometric films—on the electrical and magnetotransport properties of Mn-excess films remains insufficiently understood. In particular, the interplay between defect redistribution, oxygen vacancy dynamics, and an altered cation stoichiometry during aging requires further investigation.

In this work, we present a comparative study of accelerated aging in polycrystalline La_1−x_Sr_x_Mn_y_O_3_ thin films with two manganese concentrations: a nominally stoichiometric composition (*y* ≈ 1) and a Mn excess (*y* = 1.15). Accelerated aging was induced by thermal annealing, and the evolution of the resistivity and magnetoresistance was analyzed using the stretched-exponential relaxation model. By comparing the characteristic relaxation parameters for both compositions, this study clarifies the roles of oxygen migration and Mn-site nonstoichiometry in defect-driven aging processes and provides insight on how to improve the long-term stability of CMR-based magnetic field sensors.

## 2. Materials and Methods

Nanostructured La_1−x_Sr_x_Mn_y_O_3_ thin films with a thickness of 400 nm were deposited on polycrystalline Al_2_O_3_ (AlO) and monocrystalline LaAlO_3_ (LAO) (MTI Corporation, Richmond, CA USA) substrates via pulsed-injection metal–organic chemical vapor deposition (PI-MOCVD) [22]. Additionally, stoichiometric La_1−x_Sr_x_MnO_3_ films deposited on disordered glass-ceramics substrate (lucalox) [23], which has an amorphous phase and several crystalline phases, were also investigated. Previous studies have shown that the highest metal–insulator transition temperature in polycrystalline LSMO films is obtained for Sr concentrations in the interval 0.17 < *x* < 0.19 [24]. For this reason, films with *x* = 0.18 were selected. The precursor solution consisted of La(thd)_3_, Sr(thd)_2_, and Mn(thd)_3_ (thd = 2,2,6,6-tetramethyl-3,5-heptandionate) dissolved in toluene was prepared at the Center for Physical Sciences and Technology, Vilnius, Lithuania. Micro-injections of this solution (3 mg each) were introduced into the system at 2 Hz. Although the overall precursor concentration was kept unchanged throughout all growth runs, the relative amounts of the metal–organic compounds were varied to maintain a constant La/Sr ratio (La_0.82_Sr_0.18_) in the grown films while adjusting the *y* = Mn/(La + Sr) ratio to nominally stoichiometric and nonstoichiometric compositions. The obtained *y* values in the films were 1.05 and 1.15. This growth method enables precise control of multicomponent stoichiometry, rapid adjustment of the chemical composition, and reproducible deposition of relatively thick films with good uniformity. Moreover, compared with conventional MOCVD, PI-MOCVD ensures that the vapor phase closely reflects the initial solution through sequential microdose injection. This allows precise control of the stoichiometry, thickness, and growth rate, which is essential for multicomponent systems such as La_1−x_Sr_x_Mn_y_O_3_.

After injection, each microdose undergoes flash evaporation, i.e., rapid and nearly instantaneous vaporization due to the elevated temperature (~260 °C) and reduced pressure in the reactor (~10 Torr), forming a homogeneous vapor mixture that is transported by the Ar/O_2_ (1.9:1) carrier gas with a velocity of about 114 L/h towards the heated substrate. The substrate temperature during film deposition was kept at 750 °C. Upon completion of the process, the films were annealed in pure oxygen for 10 min at the same temperature and then cooled slowly to room temperature.

The elemental composition of the LSMO films was quantified via inductively coupled plasma high-resolution mass spectrometry (ICP-MS, Thermo Scientific Element 2) (Thermo Fisher Scientific, Bremen, Germany). Samples were fully dissolved in HNO_3_ prior to analysis. Measurements were repeated on several specimens (typically three), and the average values of *x* and *y* in La_1−x_Sr_x_Mn_y_O_3_ were obtained, with a compositional uncertainty below 1%.

The crystal structure of the films was investigated using X-ray diffraction (XRD) (SmartLab diffractometer, Osaka, Japan), while the detailed microstructure and composition analysis of the samples was characterized in cross-sectional geometry using a transmission electron microscope (TEM) (Tecnai G2 F20 X-TWIN, Amsterdam, The Netherlands).

For electrical transport and magnetoresistance measurements, Ag contacts with a Cr adhesion layer were deposited thermally and subsequently annealed at 450 °C for 1 h in an Ar atmosphere.

Accelerated aging of the LSMO films was carried out at a temperature of *T*_a_ = 100 °C in an Ar atmosphere, which was found to be optimal for manganite films [18,25]. These studies showed that maximum stability of the electrical parameters of nanostructured manganite films can be achieved by annealing them for approximately 24–28 h at this temperature. Thermal treatment at higher temperatures revealed different time-dependent relaxation behavior which was attributed to structural changes not only in the grain boundaries (oxygen depletion and diffusion) but also in the crystallites. During the initial stage of aging (up to 4 accumulated hours), the main parameter of the films—resistivity—was measured at room temperature every hour. Then, the annealing interval between measurements was chosen according to the relative resistance change. Aging was conducted in a horizontal tube furnace under continuous Ar flow. The samples (lateral size = ~0.5–1 mm) were placed in the central isothermal zone of the furnace tube (diameter = 8 cm, length = 50 cm), where the temperature distribution was uniform. Owing to the small sample dimensions relative to the furnace volume, as well as the use of a stabilized temperature regime and continuous gas flow, all samples experienced identical and uniform thermal conditions during annealing.

The temperature dependence of the resistivity *ρ*(*T*) was measured before and after accelerated aging using a closed-cycle helium cryocooler (JANIS) (Janis Research Company, Woburn, MA USA) in the range of 5–310 K and a liquid thermostat (Lauda ECO RE 420) (Lauda Scientific GmbH, Lauda-Königshofen, Germany) in the range of 255–370 K. Magnetoresistance measurements were conducted in a permanent magnetic field of up to 0.7 T at temperatures between 80 K and 300 K using an electromagnet. Measurements in pulsed magnetic fields of up to 20 T were performed in the same temperature range using a nondestructive capacitor-driven pulsed magnet installed at the Center for Physical Sciences and Technology in Vilnius, Lithuania. Several specimens (3–5) were measured to ensure reproducibility.

## 3. Results

### 3.1. Characterization of the Films

**Figure 1:**
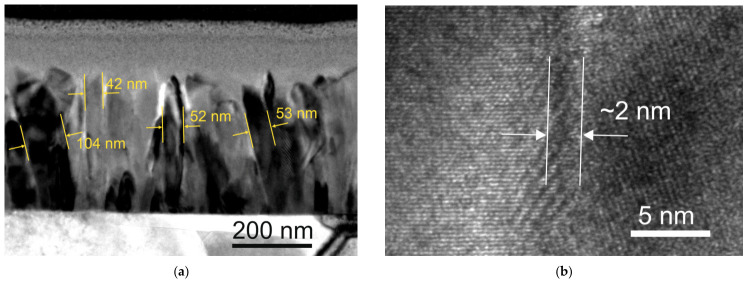
(**a**) Low-magnification cross-sectional bright-field TEM image of LSMO/AlO film with *y* = 1.15. (**b**) High-resolution TEM image of a grain boundary between two crystallites.

The study on the microstructure of the films grown with excess manganese revealed that their structure was similar to that of films with stoichiometric Mn concentration, exhibiting columnar crystallites. Low-magnification cross-sectional bright-field TEM images of the films showed that the typical column width was approximately *w* = ~40–100 nm as indicated in Figure 1a, and these columns extended throughout the entire film thickness, with their long axes oriented perpendicular to the substrate. Moreover, the contrast in the TEM images within the columns suggests that some columns were not single crystalline columns and consisted of several single-crystal slabs. The high-resolution TEM image in Figure 1b shows two single-crystal columns with a regular structure separated by an intercrystalline region, where the lattice appears distorted. The width of this region was estimated to be approximately 2 nm. Analysis of other areas of the samples showed that the width of this distorted structure ranged from 2 nm to 10 nm.

To study the distribution of chemical elements in the crystallites and grain boundary areas, energy-dispersive X-ray (EDX) spectroscopy in scanning transmission electron microscopy (STEM) was used.

**Figure 2:**
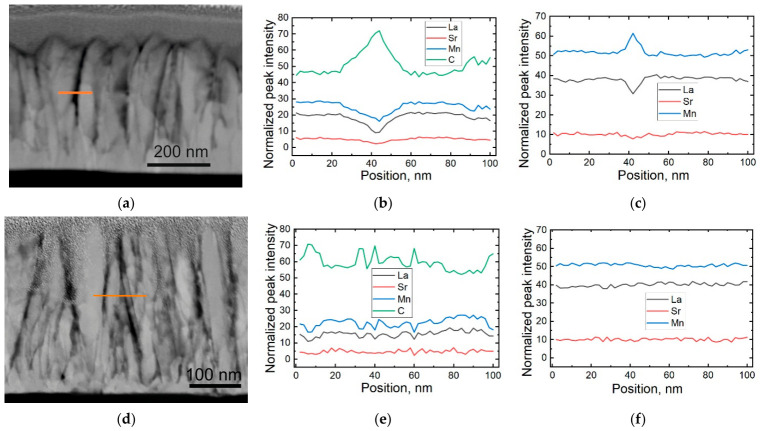
(**a**,**d**) STEM images of the LSMO/AlO film, showing the line along which elemental scanning was performed. (**b**,**e**) Peak intensities of La, Sr, Mn, and C normalized to total counts. (**c**,**f**) Peak intensities of La, Sr, and Mn normalized to total counts. (**a**–**c**) Film with *y* = 1.15. (**d**–**f**) Film with *y* = 1.05.

Figure 2a shows the STEM image of the film and the line along which the distributions of La, Sr, Mn, and C were scanned. The image contrast was influenced by the density and chemical composition of the film. The normalized profiles of these elements across the grain boundary are presented in Figure 2b. The graph shows that in the grain boundary area, the concentrations of the main film elements (La, Sr, and Mn) decreased while the carbon concentration increased. Carbon contamination is a significant issue when films are grown using the MOCVD technique, primarily due to the thermal decomposition of organic molecules [26]. During film growth, carbon is pushed from the surface of the crystallites into the grain boundary region, where it accumulates. This accumulation hinders the free migration of atoms between individual columns during film growth. As a result, after reaching a certain thickness, the diameter of the columns ceases to change. The amount of carbon pushed into the grain boundary region keeps the grain boundary material within a thickness range between 2 and 10 nm. At this particular scanning region, the width of the area with increased an carbon concentration was approximately 20 nm. In other intergranular areas, the extent of increased carbon concentration varied, typically being narrower. This was confirmed by the image of the grain boundary area shown in Figure 1b. As can be seen, the area with a disrupted structure in this part of the layer was only about 2 nm. It should be noted, however, that for nanostructured films, carbon contamination is not a significant issue, as the interior of the crystallites is not contaminated. In Figure 2c, a normalized profile of only the main elements (La, Sr, and Mn) is presented. It can be observed that at the boundary between the two crystallites, there was an increased concentration of Mn relative to La, with this increase extending over approximately 6 nm. The results obtained for the films with a nominally stoichiometric Mn concentration did not show increases in the Mn concentration in the grain boundary region (Figure 2d–f). Unfortunately, the EDX method for assessing the concentration of elements in films is not accurate enough to measure absolute concentrations and cannot provide precise stoichiometry (such as the exact values of x and y in La_1−x_Sr_x_Mn_y_O_3_) with the same level of accuracy as ICP-MS. However, EDX remains highly effective for comparative analysis. In this work, we used EDX to evaluate spatial variations in elemental distribution, particularly across grain boundaries. By comparing the normalized intensity profiles acquired under identical experimental conditions, we were able to identify relative changes in composition (e.g., enrichment or depletion of specific elements) rather than absolute concentrations. Therefore, the results presented in Figure 2c do not imply that the increased Mn concentration was observed only in the grain boundary areas. An increased Mn concentration within the crystallites of the nonstoichiometric film was proven by agreement between the ICP-MS measurements of both the polycrystalline and monocrystalline films.

**Figure 3:**
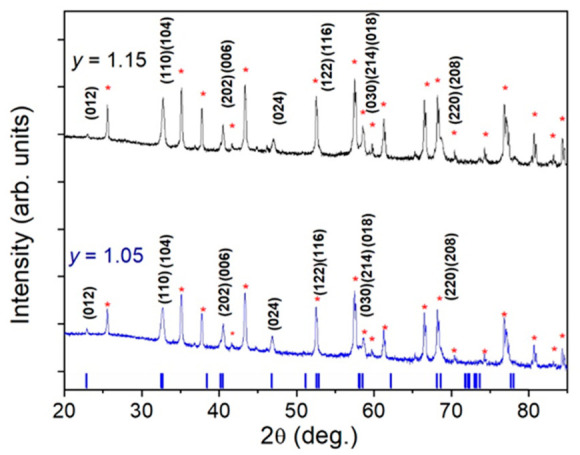
X-ray diffraction spectra for the LSMO films with *y* = 1.15 and *y* = 1.05. The stars (*) represent the characteristic peaks of the Al_2_O_3_ substrate. The sticks represent the positions of the characteristic peaks of LSMO in rhombohedral distortion obtained by modeling using the LeBail method.

The representative out-of-plane θ–2θ X-ray diffraction patterns of films with a manganese excess *y* = 1.15 and *y* = 1.05 are presented in Figure 3. The analyses of these patterns show only the characteristic peaks of the Al_2_O_3_ substrate and polycrystalline LSMO films with a perovskite-like crystal structure with rhombohedral distortions (the space group R3c). The main perovskite reflections, such as (012), (110), (104), appeared at quite similar 2θ positions in both samples. However, in the case of the *y* = 1.15 film, the peaks were slightly shifted towards higher 2θ values, indicating a reduction in the lattice parameter. This behavior can be attributed to excess Mn and a corresponding increase in the Mn^4+^ content, which had a smaller ionic radius than Mn^3+^. Additionally, the *y* = 1.15 sample exhibited broader diffraction peaks than the *y* = 1.05 sample, suggesting increased structural disorder or microstrain.

### 3.2. Accelerated Aging Influence on Resistivity

**Figure 4:**
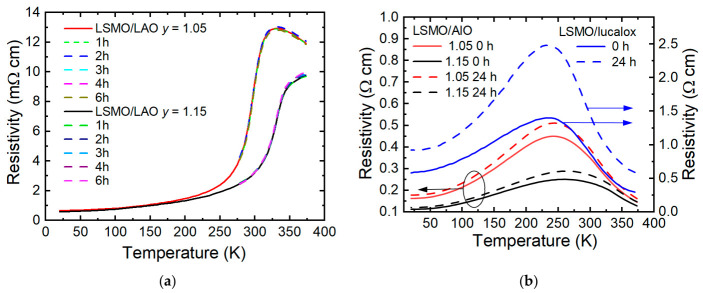
(**a**) Resistivity versus temperature dependences of LSMO films grown on monocrystalline LAO after different thermal treatment durations during accelerated aging process. (**b**) Resistivity versus temperature dependences of LSMO films grown on polycrystalline Al_2_O_3_ (left axis) and lucalox (right axis) substrates before and after 24 h of thermal treatment at 100 °C in Ar atmosphere.

Figure 4a presents the resistivity versus temperature dependences of the LSMO samples grown on monocrystalline LaAlO_3_, measured before and after different time intervals of accelerated aging. One can observe that the Mn concentration significantly affected the resistance of the grown films. When exceeding the stoichiometric Mn concentration, the resistance of the films decreased, and the maximum resistance value shifted to higher temperatures. This indicates that the excess of Mn was present not only in the grain boundary area (see Figure 2c) but also within the crystallites themselves. The same figure shows the result of annealing the films in an Ar atmosphere at 100 °C, measured after different time intervals. One can see that the resistivity as well as *T*_m_ almost did not change during the 6 h of thermal treatment. This means that under these conditions, the monocrystalline films did not change their properties regardless of chemical composition. Therefore, it was expected that the properties of individual crystallites in polycrystalline films also did not change during annealing at low temperatures (100 °C is much lower than the ~750 °C typically used in deposition or annealing [27,28]). However, the effect of annealing on the resistivity of the polycrystalline films was significant, which then can be associated with changes in the GBs.

Figure 4b shows the dependences of the resistivity on the temperature for the LSMO/AlO films with different Mn concentrations as well as the stoichiometric LSMO/lucalox film before and after thermal treatment over 24 h. One can see that all films exhibited a transition from a metal-like to an insulator-like resistivity dependence on the temperature at a certain critical temperature *T*_m_ corresponding to the resistivity maximum. It has to be noted that for the nanostructured LSMO/AlO films, *T*_m_ increased while the resistivity decreased with an increase in Mn excess. This shift can be explained by the La + Sr vacancies caused by Mn excess, acting as self-dopants.

Analysis of these results shows that the thermal treatment used to accelerate aging led to an increase in the resistivity of the films. At the same time, the temperature corresponding to the maximum resistivity *T*_m_ remained unchanged after treatment. It can also be observed that the resistivity variation was significantly smaller for the films grown on Al_2_O_3_ substrates than those grown on more disordered lucalox substrates.

**Figure 5:**
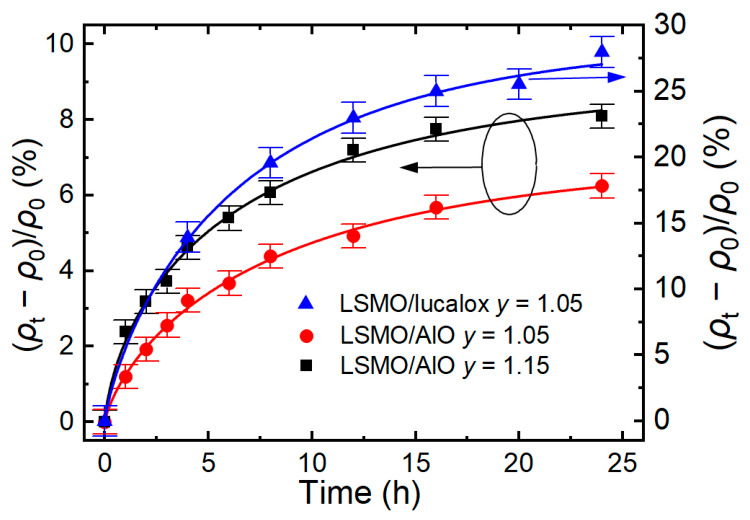
Kinetics of relative change in resistivity measured at temperature 300 K during accelerated aging process of LSMO samples with *y* = 1.05 and *y* = 1.15 grown on the Al_2_O_3_ substrates (left scale) and with a concentration of 1.05 grown on the lucalox substrate (right scale). Points—experimental results and curves—are fitted to Equation (1).

The changes in relative resistivity (*ρ*_t_ − *ρ*_0_)/*ρ*_0_ measured at 300 K during the thermal treatment are presented in Figure 5. Here, *ρ*_0_ is the initial resistivity measured before the accelerated aging process, while *ρ*_t_ is the resistivity measured after a certain treatment time *t*.

The kinetics of the resistivity changes was analyzed using a normalized relaxation function expressed as a “stretched” exponent [16,17,29]:

(*ρ*_t_ − *ρ*_0_)/*ρ*_0_ = [(*ρ*_∞_ − *ρ*_0_)/*ρ*_0_] × {1 − *exp*[−(*t*/*τ*)*^k^*]}, (1)

where τ is the characteristic time, *k* is the stretched exponent ratio, and *ρ*_∞_ is the ultimate resistivity after accelerated aging (when *t* → ∞). The fitting parameters for the films with *y* = 1.05 were (*ρ*_∞_ − *ρ*_0_)/*ρ*_0_ = 0.07, *τ* = 8.5 h, and *k* = 0.75, while for the films with *y* = 1.15, they were (*ρ*_∞_ − *ρ*_0_)/*ρ*_0_ = 0.091, *τ* = 6.8 h, and *k* = 0.68. However, it should be noted that for the films grown on lucalox substrates, the normalized resistivity change was much higher ((*ρ*_∞_ − *ρ*_0_)/*ρ*_0_ = 0.3). Such films have a more disordered structure with wider grain boundaries. As we can see, this affected not only the resistivity but also the aging process of the films. As shown in [17,18,25], the process, described by this kind of function, is typical for degradation in topologically disordered systems. The investigated nanostructured LSMO films consisted of grain columns (crystallites) having almost perfect structure and intergranular areas (grain boundaries (GBs)), with a high number of various defects created during the film deposition process (see Figure 1). Thus, the resistivity and magnetoresistive properties in these materials are mostly determined by the properties of the GB areas.

It has been shown that the relaxation mechanism depends on *k* [16,17,29]. For *k* ≥ 0.6, the hierarchically limited relaxation mechanism is most likely to occur [29]. In this model, each individual relaxation event in a disordered solid became possible only after the preceding relaxation events occurred. The fitting results indicate that for the films with Mn excess ratios *y* = 1.05 and *y* = 1.15, the resistivity relaxation during aging followed a rather similar hierarchically limited mechanism (*k* = 0.75 and *k* = 0.68, respectively).

It has to be noted that despite the change in film resistance after the accelerated aging procedure, no significant changes in the X-ray diffraction patterns were observed for the investigated films. This indicates that annealing at 100 °C did not alter the crystalline structure of the films. The results suggest that the aging process involves only weakly bound oxygen and that oxygen loss and diffusion occur mainly along grain boundaries rather than as a result of structural modification of the crystallites.

Oxygen plays the main role in the increase in resistivity during the accelerated aging process, as it participates in the double-exchange mechanism between manganese ions (Mn^3+^–O–Mn^4+^). During thermal treatment of the samples at 100 °C, only oxygen with relatively low binding energy—primarily located in disordered grain boundaries and characterized by a higher diffusion coefficient—can be released [15,30,31]. This process leads to an increase in resistivity for manganites [32,33]. The results show that the aging process of films depends rather little on the chemical composition, specifically the Mn concentration, and instead on the intercrystalline (grain boundary) structure and disorder. This is evident in the example of films grown on different types of substrates: monocrystalline (LAO), polycrystalline (Al_2_O_3_), and amorphous (lucalox).

For practical applications of manganite films as core elements of magnetic field sensors operating at room temperature, the high-temperature range is of greater importance. Therefore, the resistivity–temperature dependences of the prepared LSMO samples were analyzed in a paramagnetic state at temperatures *T* > *T*_m_. The experimental data were found to be described well by Mott’s variable range hopping (VRH) model, which is commonly used for manganite materials [34,35]. Within the framework of the double-exchange mechanism used to explain electrical transport in manganites, electrons become localized by a random spin-dependent potential above the Curie temperature, resulting in competition between the potential energy difference and the hopping distance [34,35]. According to Mott’s VRH model, the electrical resistivity for three-dimensional hopping can be expressed as follows [35,36]:

*ρ* = *ρ*_res_ *exp*(*T*_0_/*T*)^1/4^, (2)

where *ρ*_res_ is the residual resistivity and *T*_0_ is the Mott’s characteristic temperature, which can be expressed as follows:

*T*_0_ = 18/[*a*^3^*k*_B_*N*(*E*_F_)], (3)

where *a* is the localization length, *k*_B_ is the Boltzmann’s constant, and *N*(*E*_F_) is the density of states at the Fermi level.

**Figure 6:**
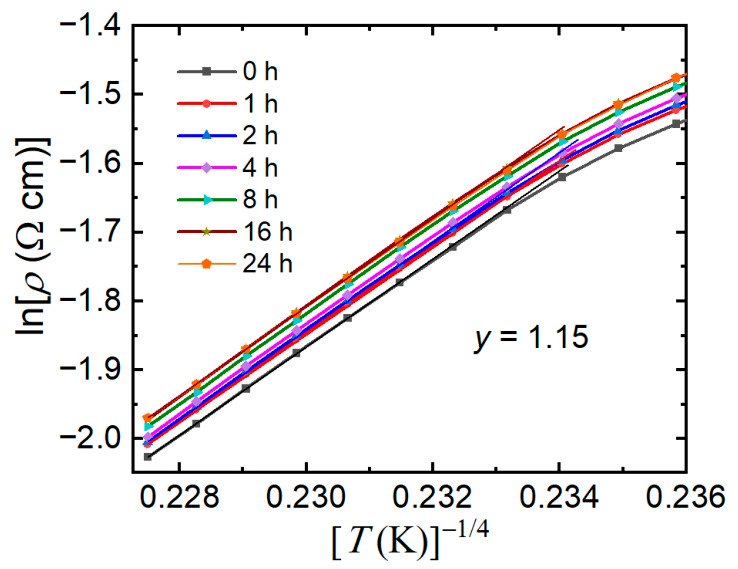
Plots of ln*ρ* versus *T*^−1/4^ for sample with *y* = 1.15 annealed at 100 °C for different accumulated annealing times.

**Figure 7:**
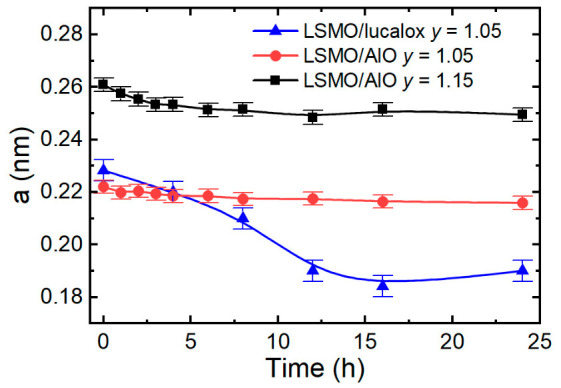
The localization length change over time during annealing of the samples with *y* = 1.05 and *y* = 1.15 procedures.

Figure 6 presents the resistivity versus temperature data at different accumulated annealing times of the sample with *y* = 1.15, fitted using Mott’s VRH model. The experimental data fit well with Equation (2) at temperatures higher than 340 K. This suggests that the conduction mechanism at temperatures higher than *T*_m_ was determined by Mott’s variable range hopping of the localized charge carriers. Figure 7 shows the localization length change during annealing of different samples. These values were calculated according to Equation (3). It has to be noted that the obtained values have no physical meaning because the localization length has to exceed the Mn^3+^-Mn^4+^ distance [35]. However, the change in the localization length with the thermal treatment time could be used for the charge carrier transport analysis. It can be seen that the highest change in localization length for both samples was achieved during the first 3 h of the annealing process. The main explanation for the decrease in localization length as well as the increase in resistance of the samples during the aging process could be based on the creation of an oxygen deficiency. Due to oxygen depletion, the double-exchange mechanism became limited, and as a result, the localization length of the wave function of the charged carriers decreased, and the resistivity increased. Similar results were obtained in our previous study on LSMO/lucalox films [18].

### 3.3. The Aging Influence on the Magnetoresistance

The accelerated aging influence on the magnetoresistance (*MR*) of the samples was investigated in low and high magnetic field ranges. The *MR* was defined as follows:

*MR* = 100·[*ρ*(*B*) − *ρ*(0)]/*ρ*(0), (4)

where *ρ*(*B*) and *ρ*(0) are resistivity values in applied magnetic field *B* and without it, respectively.

**Figure 8:**
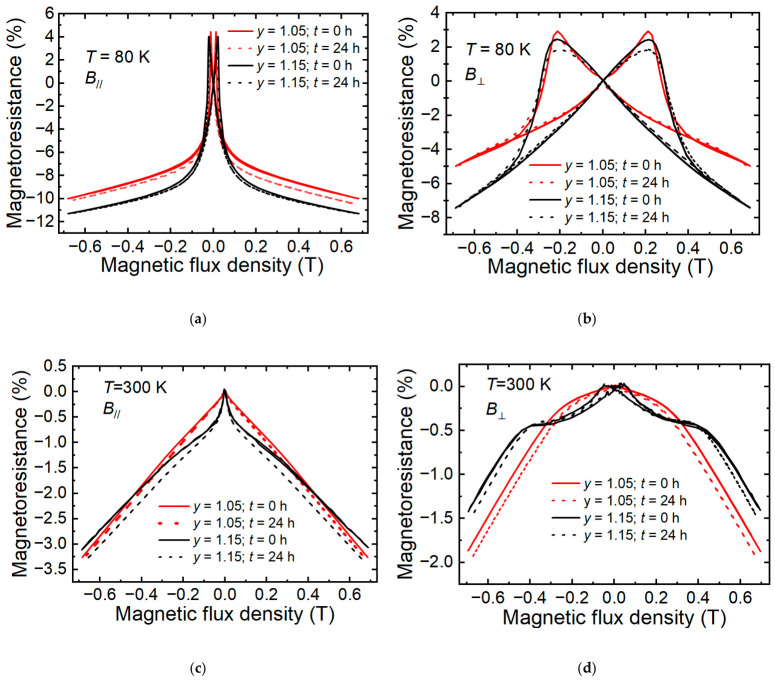
The magnetoresistance versus magnetic flux density dependencies of the samples with *y* = 1.05 and *y* = 1.15 measured at different temperatures (*T*) before (0 h) and after annealing over 24 h. (**a**) *T* = 80 K; magnetic field (*B*_//_) applied parallel to the film plane. (**b**) *T* = 300 K; magnetic field (*B*_⊥_) applied perpendicular to the film plane. (**c**) *T* = 80 K; *B*_//_. (**d**) *T* = 300 K; *B*_⊥_.

Figure 8 shows the magnetoresistance versus magnetic flux density dependencies in the low field range (*B* = 0–0.7 T) of the samples at 80 K (a,b) and 300 K (c,d) temperatures before and after 24 h of annealing. The (a,c) and (b,d) graphs present *MR* measurements performed by applying a magnetic field parallel and perpendicular to the film plain, respectively. As one can see, the low-field *MR*(*B*) dependences are typical for such films at temperatures corresponding to both ferromagnetic (80 K) and paramagnetic (300 K) states [37]. Namely, when the magnetic field was oriented parallel to the surface of the films, in a magnetic field < 0.2 T, we observed a so-called low field magnetoresistance (LFMR) effect with characteristic hysteresis. The observation of LFMR in polycrystalline manganite films is attributed to the spin-polarized tunneling of charge carriers across grain boundaries [38,39], whose underlying mechanism has been extensively illustrated schematically in the literature [40,41]. The hysteresis effect for *MR* when the magnetic field is perpendicular to the film plain (Figure 8b) was more pronounced, and this is related to the demagnetization effect (“shape” effect) which is observed in thin magnetic films. At the same time, the difference in *MR* for the films with different Mn concentrations was caused by the difference in the maximum resistance temperature *T*_m_, which was higher for the films with *y* = 1.15. Moreover, it can be clearly seen that accelerated aging almost did not influence the *MR* dependence versus the magnetic field at 80 K for both the *B*_//_and *B*_⊥_ cases. This is in contrast to our previous studies [18,25], in which accelerated aging of manganite films grown on lucalox substrates led to a strong decrease in the demagnetization field when the magnetic field was applied perpendicular to the film surface (*B*_⊥_). This magnetoresistance behavior was explained by the weakening of the magnetic coupling between the crystallites after annealing. Due to oxygen loss in the grain boundaries, the long-range interaction between the crystallites decreases within the plane of the film, which causes the weaker demagnetization effect [18].

The present results on LSMO/AlO films show that the demagnetization field did not change after thermal treatment (Figure 8b). This could be explained by the larger crystallites and thinner GBs regions, resulting in a less disordered structure for the films, as can be seen from lower resistivity compared with the LSMO/lucalox films (see Figure 4b). Moreover, after the 24-h aging process, the relative resistivity change in LSMO/AlO was only (6–8)%, while for LSMO/lucalox, it reached 28%. Since the resistivity of polycrystalline films is mainly controlled by the resistivity of the grain boundaries, it can be concluded that the oxygen loss from the grain boundaries has a weaker influence on the long-range interaction between the crystallites in less disordered LSMO/AlO films.

Insignificant changes after treatment of the LSMO/AlO films at 100 °C were also observed at room temperatures (see Figure 8c,d).

The obtained results for magnetoresistance change during accelerated aging are important for the development of magnetic field sensors, which can operate in high magnetic fields.

**Figure 9:**
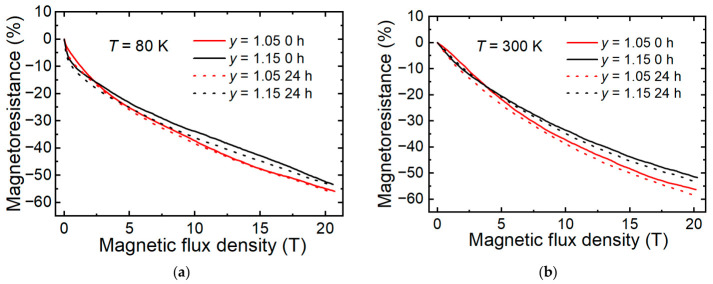
*MR* versus *B* dependences of LSMO/AlO films with *y* = 1.05 and *y* = 1.15, measured at (**a**) 80 K and (**b**) 300 K temperatures before and after accelerated aging process.

Figure 9 shows the influence of accelerated aging on the high-field MR(*B*) dependences for the samples with *y* = 1.05 and *y* = 1.15. For both compositions, at low (80 K) and high (300 K) temperatures, the change in magnetoresistance across the entire magnetic field range during accelerated aging remained quite small (<1%).

This behavior can be explained by the double-exchange mechanism governing electrical transport in manganites [1]. In these materials, electron transport is spin-polarized and occurs via electron transfer from Mn^3+^ to Mn^4+^ ions through an intermediate oxygen ion. When the spins of neighboring Mn ions are misaligned, electron transfer becomes less probable, and charge carrier mobility decreases. The application of a magnetic field aligns the Mn core spins, thereby reducing the resistivity.

Thermal treatment of the films at relatively low temperatures (100 °C) primarily activates weakly bound oxygen depletion and its diffusion along grain boundaries [30,31]. These processes reduce the number of Mn^3+^–O–Mn^4+^ hopping centers participating in the double-exchange transport mechanism. Consequently, the overall resistivity increases, while the ratio *ρ*(*B*)/*ρ*(0), and therefore the magnetoresistance, remain nearly unchanged.

The observed stability of *MR* during accelerated aging across the entire magnetic-field range up to 20 T is a promising result for the development of high-pulse magnetic field sensors with long-term stability in their parameters.

## 4. Conclusions

Accelerated aging of nanostructured nonstoichiometric La_1−x_Sr_x_Mn_y_O_3_ thin films at 100 °C (Ar atmosphere) resulted in a moderate resistivity increase governed by grain boundary defect relaxation. After 24 h, the films grown on polycrystalline Al_2_O_3_ exhibited a resistivity increase of 6–8%, while the films on amorphous lucalox showed a much larger change of ≈28%, and the films on monocrystalline LaAlO_3_ remained stable. The aging kinetics followed a stretched-exponential law, with similar parameters for both compositions (*τ* ≈ 6.8–8.5 h, *k* ≈ 0.68–0.75), indicating weak dependence on Mn-nonstoichiometry (*y* = 1.05–1.15). The metal–insulator transition temperature *T*_m_ was unchanged by aging. Importantly, the magnetoresistance remained highly stable, with changes of <1% up to 20 T at 80–300 K. These results show that a Mn excess (*y* = 1.15) reduces resistivity and extends the useful temperature range without compromising long-term stability, confirming the suitability of these films for CMR-based magnetic field sensors.

## Data Availability

The original contributions presented in this study are included in the article material. Further inquiries can be directed to the corresponding author.
